# The effect of cyclic martensitic transformations on diffusion of cobalt atoms in Fe-18wt.%Mn-2wt.%Si alloy

**DOI:** 10.1186/s11671-015-0884-5

**Published:** 2015-04-14

**Authors:** Vitaliy E Danilchenko, Vladimir F Mazanko, Alexander V Filatov, Viktor E Iakovlev

**Affiliations:** G.V. Kurdyumov Institute for Metal Physics, NAS of Ukraine, Vernadsky Blvd. 36, Kyiv, 03142 Ukraine

**Keywords:** Martensitic transformation, Diffusion, Packing defects, Dislocations density, Phase hardening

## Abstract

Diffusion characteristics of cobalt atoms were investigated using radioactive isotope method in phase-hardened Fe-18wt.%Mn-2wt.%Si alloy. The observed significant increase of diffusion coefficient of cobalt atoms under the cyclic γ-ε-γ martensitic transformations was due to the action of two independent mechanisms - an athermal one and a thermally activated one. The first one arose from the direct γ-ε and the reverse ε-γ transformations with corresponding direct and reverse lattice shears during alternating stresses and simultaneous lattice restructuring. Another mechanism arose under the diffusion annealing of the phase-hardened alloy.

## Background

Phase transformations are the ones that most significantly affect the diffusion properties of interstitials and substitution atoms in metastable alloys [1]. The extent and the features of such influence depend on the alloys structural state that arises due to phase transformations. As a result of phase and structural transformations in metals and alloys accompanied by a volume effect, a system of lattice defects was generated that intensifies the diffusion processes and boosts the mass transfer of atoms on macroscopic distances. Martensitic γ-α-γ transformations in Fe-Ni alloys, implemented by the shear diffusionless mechanism, essentially accelerate the diffusion of atoms in the reverted phase. The diffusion coefficient of alloying atoms depends essentially on cooling and heating conditions required for the direct γ-α and the reverse α-γ martensitic transformations, respectively [2].

As a result of γ-ε-γ transformations in Fe-Mn alloys, the dislocation density increases in the reverted austenitic phase by an order of magnitude [3]. Such difference in the increase is caused by different values of the volume effect of the γ-α and γ-ε transformations (3% to 4% and 1.75%, respectively). In a Fe-Mn reverted austenite with low energy of packing defects, cyclic γ-ε-γ transformations cause accumulation of random packing defects but do not lead to considerable structure nanofragmentation and additional subboundaries generation. The essential difference in the structural state and the defect rate of structure elements formed as a result of γ-α-γ and γ-ε-γ transformations, point to the necessity for an additional study of the γ-ε-γ transformations influence on diffusion in alloys with low energy of packing defects. We know only one experimental study [4] of the influence of cyclic γ-ε-γ transformations in Co- and Fe-Mn alloys on the diffusion parameters of substitution atoms. There, an essential increase of their diffusivity has been detected. Nevertheless, in [4] only an integrated result of the intensification of the diffusion processes has been obtained which is due to the overall influence of the factors of martensitic transformation. Our work is the first attempt to distinguish between the contribution to the intensification of diffusion processes by really martensitic γ-ε-γ transformations and by structural defects formed as a result of diffusion structure changes during such transformations.

## Methods

Our investigations were carried out in Fe-18wt.%Mn-2wt.%Si alloy with high completeness of γ-ε transformation (more than 90% of martensitic ε-phase). This has enabled us to reach a high scale phase hardening by cyclic γ-ε-γ transformations.

The direct, γ-ε transformation in the alloy, occurred as the result of cooling in liquid nitrogen, and the reverse, ε-γ one, took place during consequent heating in a salt bath (55%NaNO_3_ + 45%KNO_3_) at the temperature of 380°C. The temperature points of the investigated alloy were: *Ms* = 80°C, *Mf* = −110°C, *As* = 220°C, *Af* = 380°C.

The cooling and the heating rates during the transformations were made as high as possible and reached 20°/s and 80°/s, respectively. Such mode of thermocycling inhibited relaxation processes and provided the effective accumulation of structural defects as a result of the direct and the reverse transformations. Such defects are capable to influence significantly on diffusion processes in phase hardened alloys. Really, in [5] it has already been noted that a noticeable increase of the diffusion coefficient in iron and thallium was found only by reaching some critical rate of thermocycling. After γ-ε-γ transformations, homogenizing annealing of phase hardened alloys was carried out at 250°C and 325°C.

Phase analysis was performed on automatic X-ray diffractometer DRON-3 under monochromatic Fe-radiation. The amount of ε-phase was measured by the integrated intensity of diffraction reflexes (111)γ and (002)ε ratio [6]:

1 $$ {M}_{\varepsilon }=\frac{100\%}{1+0.27\frac{{I_{(111)}}_{\gamma }}{{I_{(002)}}_{\varepsilon }}} $$

The use of the specified reflexes from austenite and ε-martensite areas, which were parallel in accordance with orientation correlations between FCC and HCP lattices, has enabled us to calculate the amount of ε-phase without a reduction in the measurement precision that could arise in case of a texture appearance under the thermocycling. The amount of martensitic phase was measured with the accuracy of 0.5%.

The diffusion characteristics were determined by a radioactive method. A layer with radioactive isotope ^60^Co was deposited on one of the austenitic alloy surfaces. The thickness of the isotope was less than 0.5 microns and β-activity was (5 × 10^3^ ± 50) pulses/min. The concentration distribution of cobalt in depth after the cyclic martensitic γ-ε-γ transformations and diffusion annealing at temperatures 250°C and 325°C was determined by removing the layers of the sample of the alloy and measuring the integral radioactivity of the rest of the sample. The thickness of each layer was determined by weighing on microanalytical scales and calculating with the formula:

2 $$ \varDelta h=\frac{h_0}{P_0}\varDelta P, $$

where *h*_0_ and *P*_0_ are respectively the thickness and the weight of the initial sample, Δ*h* and Δ*P* are the thickness and the weight of the removed layer.

To calculate the diffusion coefficient, we used the following formula (from Fick’s first law) [7]:

3 $$ D=-\frac{1}{4\tau \cdot tg\alpha } $$

where *tg*(α) was determined from the graph:

4 $$ f\left({x}_n^2\right)= \lg \left(\frac{\partial {I}_n}{\partial {x}_n}\right) $$

and *t* is the diffusion annealing time, *x* is the penetration depth of the isotope into the sample, *I* is the intensity of the isotope at a certain depth. The measurement accuracy of the diffusion coefficient was 20%.

## Results and discussion

**Figure 1:**
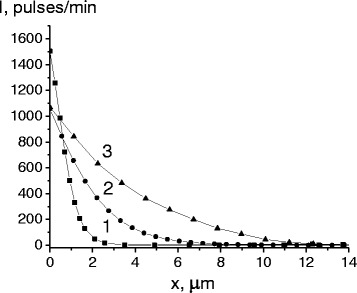
Concentration distribution of the ^60^Co radioisotope after 1, 50, and 200 cycles of transformation. Concentration distribution of the ^60^Co radioisotope in reverted austenite after 1 (1), 50 (2), and 200 (3) cycles of transformation.

The analysis of the distribution of cobalt atoms in depth has showed that, as a result of cyclic γ-ε-γ transformations without additional diffusion annealing, they penetrate into the alloy volume to certain depths. After the first cycle of transformations, the penetration depth of cobalt atoms was already 3.5 microns (Figure 1, curve 1). Increasing the number of cycles led to further penetration of cobalt atoms. After 50 and 200 cycles, the depths were 7 and 13 microns, respectively (Figure 1, curves 2 and 3).

We could not calculate the diffusion coefficient of cobalt using diffusion profiles because we had no way to determine the time length of the cobalt diffusion penetration into the bulk of the alloy, which is obviously comparable with the time of martensitic transformation. It should be considered that the mechanism of transfer of cobalt atoms is connected with actual structural f.c.c. - h.c.p. - f.c.c. restructuring and related resulting stress field due to changes in the size and volume of crystals that turn. The increase of penetration depth under repeated cycles indicated the possibility of accumulation of internal stresses that consistently stimulated further diffusion of cobalt atoms. Obviously, this mechanism of moving substitution atoms can be considered mainly athermal. Previously, it was shown that an athermal mechanism of substitution and interstitial atom mass transfer was realized by explosive kinetics of γ-α transformation in iron alloys [8,9].

Under γ-ε-γ transformations, in local areas of alloy, internal tensile and compressive stresses cyclically occurred, in accordance with the character of the direct γ-ε (increasing of specific volume) and reverse ε-γ (decreasing of specific volume) transformations. Stretched regions occurred as a result of the direct γ-ε transformation at low temperatures, when the relaxation processes were inhibited and internal stresses did persist. This created the conditions for the transport of cobalt atoms into such areas. Obviously, the reverse ε-γ transformation created more weak conditions for the migration of substitution atoms under a thermal mechanism.

In order to detect the thermally activated diffusion part in phase-hardened Fe-18wt.%Mn-2wt.%Si alloy, we subsequently conducted an investigation of isothermal annealing influence on the parameters of the diffusion of cobalt. After γ-ε-γ transformations, we additionally annealed the phase-hardened specimens for 2500 hours at 250°C and 325°C, thus realizing the diffusion annealing cycle for further stimulation of the diffusion of cobalt atoms. The annealing time at maximum temperature under cycling was rather small compared to the additional time of the diffusion annealing. To avoid relaxation processes in the reverted austenite, we prevented overheating above the temperature at the final point of inverse transformation. After thermal cycling and additional isothermal annealing, the amount of ε-phase in the samples was over 90%. This indicated that the diffusion parameters of the phase-hardened alloy related mainly to ε-phase.

**Figure 2:**
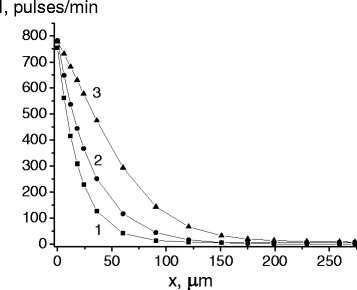
Concentration distribution of the ^60^Co radioisotope after γ-ε-γ transformations and additional annealing. Concentration distribution of the ^60^Co radioisotope in reverted austenite after 1 (1), 100 (2) and 500 (3) cycles of transformation and additional annealing at 325°C.

Diffusion annealing of the phase-hardened alloy led to the penetration of cobalt atoms to macroscopic distances. Thus, for example, after the first and the hundredth cycles the penetration depth was respectively 3.5 and 12 microns, whereas after following annealing at 250°C the penetration depth increased up to 80 and 133 microns. Increasing of annealing temperature to 325°C caused an additional penetration of tracer atoms into the alloy (Figure 2).

**Figure 3:**
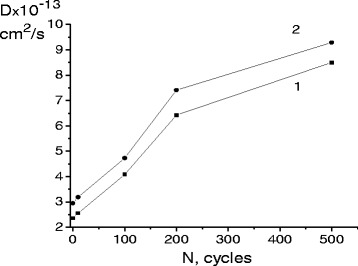
The diffusion coefficient of cobalt change as a function of the number of γ**-**ε**-**γ cycles**.** The diffusion coefficient of cobalt change as a function of the number of γ-ε-γ cycles after diffusion annealing at 250°C (1) and 325°C (2).

Calculations have shown (Figure 3) that after the first cycle of transformations the diffusion coefficient of cobalt D at a temperature of 250°C was equal to 2.36 × 10^−13^ cm^2^/s. Increasing the number of γ-ε-γ cycles has led to a monotonic increase of the parameter D. As a result of 500 cycles of transformations, the parameter D increased about 3.6 times compared to that of the first cycle.

Therefore, a significant diffusion intensification of cobalt atoms under cyclic γ-ε-γ transformations is associated with the effect of two independent mechanisms - an athermal and a thermally activated one. The first mechanism is determined by the movements of atoms in the field of internal stresses that arise as a result of the direct γ-ε transformations.

One should accept that the main mechanism of diffusion intensification due to the thermal activation is the acceleration of diffusion in dislocations. In addition, one should take into account that the low-angle subboundaries may be considered as polygonal dislocation walls. The effect of chaotic packing defects on diffusion parameters can be also indirectly associated with the influence of dislocations, because chaotic packing defects can be represented as a set of two partial dislocations with a layer of packing defects between them.

## Conclusions

The observed significant increase of diffusion coefficient of cobalt atoms under the cyclic γ-ε-γ martensitic transformations in Fe-18wt.%Mn-2wt.%Si alloy was due to the action of two independent mechanisms - an athermal one and a thermally activated one. The first one arose from the direct γ-ε and the reverse ε-γ transformations with corresponding direct and reverse lattice shears during alternating stresses and simultaneous lattice restructuring. Another mechanism arose under the diffusion annealing of the phase-hardened alloy. As a result of thermal cycling, the following defects of the crystal structure accumulated in the lattice: dislocations, low-angle subboundaries, the random packaging defects, all of them being the ways of the diffusion acceleration. With increasing the degree of the phase-hardening (up to 100 γ-ε-γ cycles), the penetration depth of atoms of the isotope increased twice and the diffusion coefficients after the first cycle and 100 cycles were equal to 2.95 × 10^−13^ and 9.29 × 10^−13^ cm^2^/s respectively.

The crystal structure defects formation in metastable alloys under the cyclic martensitic γ-ε-γ transformations and the resulting significant increase of the diffusion coefficient of substitution atoms at low temperatures opens up new opportunities for creating more intensive methods of chemical and thermal treatment. Due to the preliminary phase hardening, the temperature of the surface metallization of metastable iron-manganese alloys can be reduced by several hundred degrees.
